# The yeast checkpoint kinase Dun1p represses transcription of *RNR* genes independently of catalytic activity or Rad53p during respiratory growth

**DOI:** 10.1016/j.jbc.2025.108232

**Published:** 2025-01-27

**Authors:** Shreya Nagar, Riddhi Mehta, Pritpal Kaur, Fatema Zohra Sadia, Suprataptha Reddy, Olasubomi R. Olorunnimbe, Ivana Vancurova, Ales Vancura

**Affiliations:** Department of Biological Sciences, St John’s University, Queens, New York, USA

**Keywords:** cell metabolism, checkpoint control, DNA damage response, gene transcription, ribonucleotide reductase, *Saccharomyces cerevisiae*, checkpoint kinases

## Abstract

One of the key events in DNA damage response is activation of checkpoint kinases leading to activation of ribonucleotide reductase (RNR) and increased synthesis of deoxyribonucleotide triphosphates (dNTPs) required for DNA repair. Among other mechanisms, the activation of dNTP synthesis is driven by derepression of genes encoding RNR subunits *RNR2*, *RNR3*, and *RNR4*, following checkpoint activation and checkpoint kinase Dun1p-mediated phosphorylation and inactivation of transcriptional repressor Crt1p. We report here that in the absence of genotoxic stress during respiratory growth on nonfermentable carbon source acetate, inactivation of checkpoint kinases results in significant growth defect and alters transcriptional regulation of *RNR2-4* genes and genes encoding enzymes of the tricarboxylic acid and glyoxylate cycles and gluconeogenesis. Dun1p, independently of its kinase activity or signaling from the upstream checkpoint kinase Rad53p, represses *RNR2*, *RNR3*, and *RNR4* genes by maintaining Crt1p occupancy in the corresponding promoters. Consistently with the role of dNTPs in the regulation of mitochondrial DNA copy number, *DUN1* inactivation elevates mitochondrial DNA copy number in acetate-grown cells. Together, our data reveal an unexpected role for Dun1p in transcriptional regulation of *RNR2-4* and metabolic genes during growth on nonfermentable carbon source and suggest that Dun1p contributes to transcription regulation independently of its kinase activity as a structural component by binding to protein(s) involved in gene regulation.

Both exogenous and endogenous factors can generate genotoxic stress and cause damage to cellular DNA (1, 2, 3, 4). Since maintenance of genome stability is crucial for survival, cells have evolved DNA damage response (DDR), a set of highly conserved mechanisms to sense and signal damaged DNA (3, 4, 5). Major part of DDR is coordinated by DNA damage checkpoint (DDC) (1, 2, 3). In addition to DDC, eukaryotic cells have also DNA replication checkpoint (DRC) that is distinct from the DDC and specifically signals slowly progressing or arrested replication forks (3, 6, 7). DDR involves stalling or arrest of the cell cycle, initiation of DNA repair, and altered regulation of transcription, translation, and the ubiquitin-proteasome system (1, 3, 4, 8, 9, 10). As DNA damage may lead to replication stress and replication stress may lead to DNA damage, DDC and DRC extensively overlap. A cascade of protein kinases known as the “checkpoint kinases” is the key component of both DDC and DRC (5, 10, 11, 12, 13). Upon DDC or DRC activation, the sensor kinases (ATM/ATR in mammals, Tel1p/Mec1p in budding yeast) become active and phosphorylate the effector kinases (CHK1 and CHK2 in mammals, Chk1p, Rad53p, and Dun1p in budding yeast).

One of the key outcomes of the DRC and DDR in yeast is the enlargement of the deoxyribonucleoside triphosphate (dNTP) pools, which is a prerequisite for normal progression through the S phase as well as for effective DNA repair (14, 15). The rate-limiting step of dNTP synthesis is the reduction of ribonucleoside diphosphates into corresponding deoxyribonucleoside diphosphates, catalyzed by ribonucleotide reductase (RNR) (16). In most eukaryotes, RNR enzymes are α2β2 heterotetramers, in which α2 homodimer and β2 homodimer represent the large and small subunits, respectively. In yeast, however, the small subunit is a heterodimer of Rnr2p and Rnr4p; the large subunit is a homodimer of Rnr1p. The catalytic site is contained within the large subunit of both mammalian and yeast RNR enzymes. In *Saccharomyces cerevisiae*, the large subunit is encoded by *RNR1* and *RNR3* genes. *RNR1* is an essential gene and the primary functional form of the large subunit, while *RNR3* is not essential. Despite its lesser activity, overexpression of *RNR3* can rescue the lethality of *rnr1*Δ cells (14, 16). Both mammalian and yeast *RNR* genes are regulated transcriptionally, and the enzymes are regulated allosterically (17, 18, 19, 20, 21). In yeast, transcription of *RNR2*, *RNR3*, and *RNR4* genes is induced following checkpoint activation and Dun1p-mediated phosphorylation and inactivation of transcriptional repressor Crt1p (22).

Budding yeast *S*. *cerevisiae* preferentially uses glucose as a carbon source but is also able to utilize nonfermentable carbon sources, such as acetate (23). Growth on a nonfermentable carbon source results in metabolic reprogramming and dramatic increase of transcription of genes required for tricarboxylic acid (TCA) cycle, electron transport chain (ETC), oxidative phosphorylation, glyoxylate cycle, and gluconeogenesis (23). We report here that this transcription reprogramming requires checkpoint kinases. Our results also show that unlike in glucose-grown cells, in acetate-grown cells in the absence of genotoxic stress, Dun1p is required for repression of *RNR2*, *RNR3*, and *RNR4* genes by maintaining Crt1p occupancy in the corresponding promoters. The repression of *RNR2-4* genes and activation of gluconeogenetic and glyoxylate cycle genes in acetate-grown cells does not require Dun1p′s kinase activity or activation of Dun1p by the upstream kinase Rad53p. Cumulatively, our results reveal a new role for Dun1p and suggest that checkpoint kinases may play roles that are independent of their kinase activities.

## Results

### Checkpoint kinases are required for normal growth on nonfermentable medium

**Figure 1:**
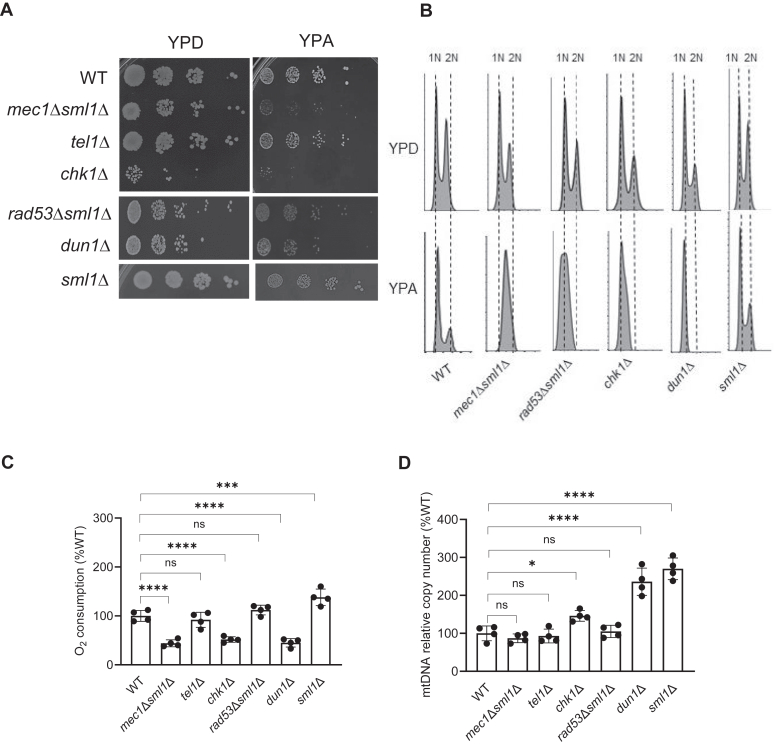
**Checkpoint kinases are required for normal growth on nonfermentable carbon source acetate**. *A*, inactivation of checkpoint kinases results in slow growth on acetate (YPA). 10-fold serial dilutions of wildtype (WT) (W303-1a), *mec1*Δ*sml1*Δ (SN117), *tel11*Δ (SN159), *chk1*Δ (SN136), *rad53*Δ*sml1*Δ (LG606), and *dun1*Δ (SN141) cells were spotted onto YPD and YPA plates and grown for 48 h and 96 h, respectively. *B*, *rad53*Δ*sml1*Δ, *chk1*Δ, and *dun1*Δ cells growing asynchronously in YPA medium accumulate in G1 phase and *mec1*Δ*sml1*Δ cells accumulate in S phase. Cells were fixed and the DNA content was measured by flow cytometry. (*A*, *B*) typical results from three independent experiments are shown. *C*, *D*, relative cellular oxygen consumption rate (*C*) and relative mtDNA copy number (D). (*C*, *D*) The data are means ± SD from four biologically independent experiments. ∗*p* < 0.05, ∗∗∗*p* < 0.001, and ∗∗∗∗*p* < 0.0001 by one-way ANOVA and Dunnett's multiple comparisons test. mtDNA, mitochondrial DNA; YPD, glucose-containing medium.

Our previous results indicated that simultaneous inactivation of ETC and *DUN1* results in a synthetic growth defect even in glucose containing medium when ETC is not required for viability (24). To explore the role of Dun1p and other checkpoint kinases during aerobic metabolism, we assessed growth of mutants with inactivated checkpoint kinases on acetate containing medium, when ETC is essential. While on glucose-containing medium (YPD), only growth of *chk1*Δ mutant was slower than growth of wildtype (WT) strain, on acetate-containing medium (YPA), all checkpoint kinase mutants grew slower than WT cells. In fact, the growth of *mec1*Δ*sml1*Δ and *chk1*Δ strains was barely noticeable after 96 h (Fig. 1*A*). To determine whether inactivation of any of the checkpoint kinases affects the distribution of cells throughout the cell cycle in YPD and YPA media, we determined by flow cytometry the corresponding cell-cycle profiles (Fig. 1*B*). While all checkpoint mutants grown in YPD show relatively normal cell cycle profiles with characteristic distribution into 1N and 2N populations, *rad53*Δ*sml1*Δ, *chk1*Δ, and *dun1*Δ cells grown in YPA medium appear to spend significantly more time in G1 phase of the cell cycle with 1N DNA content. In contrast, *mec1*Δ*sml1*Δ cells accumulate during S phase with DNA content between 1N and 2N. WT cells grown in YPA medium display distribution in 1N and 2N populations; however, majority of the WT cells are in the G1 phase of the cell cycle with 1N DNA content. Increased fraction of cells in G1 phase is typical for growth on suboptimal carbon source, such as acetate, when cells need additional time for biosynthetic processes to be able to pass the Start point of the cell cycle (25, 26, 27, 28, 29).

Since growth on nonfermentable carbon sources, such as acetate, requires mitochondrial ETC, we measured oxygen consumption, the readout for ETC activity, in mutants with inactivated checkpoint kinases. The oxygen consumption was significantly decreased in *mec1*Δ*sml1*Δ, *chk1*Δ, and *dun1*Δ cells and was not significantly affected in *tel1*Δ and *rad53*Δ*sml1*Δ cells (Fig. 1*C*). The *sml1*Δ strain was included as a positive control, since it has elevated oxygen consumption due to increased levels of dNTPs and mitochondrial DNA (mtDNA) copy number (24, 30). Since the ETC activity requires mtDNA and mtDNA copy number typically correlates with ETC activity, we determined the mtDNA copy number in the checkpoint kinase mutants (Fig. 1*D*). The mtDNA copy number did not significantly differ in *mec1*Δ*sml1*Δ, *tel1*Δ, and *rad53*Δ*sml1*Δ strains but was significantly elevated in *chk1*Δ and particularly *dun1*Δ cells (Fig. 1*D*). This finding for *dun1*Δ cells is quite surprising, since mtDNA copy number correlates with dNTP synthesis (31) and Dun1p upregulates dNTP synthesis by regulating Sml1p and Crt1p (22, 32). Inactivation of Dun1p would be thus expected to lower the mtDNA copy number.

### Checkpoint kinase mutants display altered mRNA levels of TCA cycle, ETC, gluconeogenesis, and glyoxylate cycle genes

**Figure 2:**
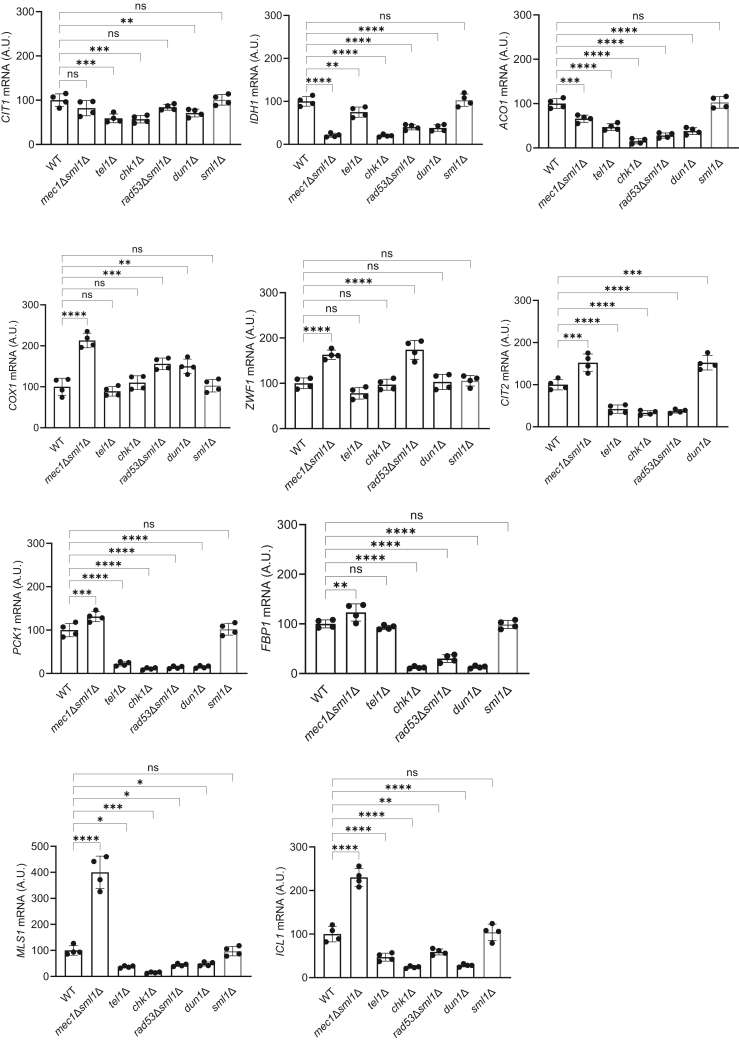
**Inactivation of checkpoint kinases alters transcriptional regulation of metabolic genes**. mRNA levels of *CIT1*, *IDH1*, *ACO1*, *COX1*, *ZWF1*, *CIT2*, *PCK1*, *FBP1*, *MLS1*, and *ICL1* in wildtype (WT), *mec1*Δ*sml1*Δ, *tel11*Δ, *chk1*Δ, *rad53*Δ*sml1*Δ, and *dun1*Δ cells. The results are shown as arbitrary units (A.U.), and the mean for WT cells was set as 100 A U. The data are means ± SD from four biologically independent experiments. ∗*p* < 0.05, ∗∗*p* < 0.01, ∗∗∗*p* < 0.001, ∗∗∗∗*p* < 0.0001 by one-way ANOVA and Dunnett's multiple comparisons test.

Since growth on a nonfermentable carbon source requires transcriptional reprogramming of metabolism, we hypothesized that the slow growth of the checkpoint kinase mutants is due to altered transcriptional regulation of genes required for growth on acetate. To test this possibility, we determined mRNA levels of genes encoding enzymes of the TCA cycle, ETC, glyoxylate cycle, gluconeogenesis, and pentose phosphate pathway (PPP) in WT, *mec1*Δ*sml1*Δ, *tel1*Δ, *chk1*Δ, *rad53*Δ*sml1*Δ, and *dun1*Δ cells grown in YPA medium (Fig. 2). mRNA levels of TCA cycle genes *CIT1*, *IDH1*, and *ACO1* were decreased in all checkpoint mutants, with the exception of *CIT1* in *rad53*Δ*sml1*Δ cells. Conversely, mRNA levels of ETC gene *COX1* were elevated in *mec1*Δ*sml1*Δ, *rad53*Δ*sml1*Δ, and *dun1*Δ cells. The mRNA level of *ZWF1*, encoding a PPP enzyme, was elevated in *mec1*Δ*sml1*Δ and *rad53*Δ*sml1*Δ cells and unchanged in the other checkpoint mutants. The mRNA level of *CIT2*, a retrograde (RTG) pathway gene, was elevated in *mec1*Δ*sml1*Δ and *rad53*Δ*sml1*Δ cells and decreased in the other checkpoint mutants. mRNA levels of *PCK1* and *FBP1*, genes encoding gluconeogenesis enzymes, were significantly decreased in *tel1*Δ, *chk1*Δ, *rad53*Δ*sml1*Δ, and *dun1*Δ cells with the exception of *FBP1* in *tel1*Δ cells, where they were unchanged. In contrast, in *mec1*Δ*sml1*Δ cells, *PCK1* and *FBP1* levels were elevated. The mRNA levels of *MLS1* and *ICL1*, genes encoding glyoxylate cycle enzymes, were elevated 4-fold and 2.5-fold in *mec1*Δ*sml1*Δ cells and significantly downregulated in all other checkpoint mutants.

In general, these results show that the transcription program of key metabolic pathways required for growth on nonfermentable carbon source acetate is significantly deregulated in all checkpoint mutants (Fig. 2). The most significant changes are found in transcription of genes encoding enzymes of the TCA cycle, glyoxylate cycle, and gluconeogenesis. The regulation of glyoxylate cycle and gluconeogenesis genes displays similar pattern and is of particular interest. While the mRNA levels of gluconeogenesis genes *FBP1* and *PCK1* and glyoxylate cycle genes *MLS1* and *ICL1* are upregulated in *mec1*Δ*sml1*Δ cells, they are downregulated in *tel1*Δ, *chk1*Δ, *rad53*Δ*sml1*Δ, and *dun1*Δ cells (Fig. 2). The key transcription factor of gluconeogenesis and glyoxylate cycle genes is Cat8p (23, 33, 34). Interestingly, Cat8p was found to translocate to the nucleus during replication stress (35). It is thus possible that checkpoint kinases regulate function of Cat8p. Remarkably, inactivation of Mec1p has the opposite effect on transcription of gluconeogenesis and glyoxylate cycle genes than inactivation of other checkpoint kinases (Fig. 2). If Cat8p is indeed the target of checkpoint kinases, it would mean that Mec1p opposes and other checkpoint kinases promote the function of Cat8p in transcription of gluconeogenesis and glyoxylate cycle genes.

In addition, inactivation of Mec1p likely changes distribution of carbon flux between catabolism and anabolism. Since TCA and glyoxylate cycles share a number of common intermediates and serve primarily catabolism and anabolism, respectively, their relative activities are important for determining carbon flux through these main branches of metabolism. The main function of the TCA cycle is to produce NADH molecules for ETC and ATP production through conversion of acetyl-CoA to CO_2_. The main function of glyoxylate cycle is to generate 4-carbon intermediates from two molecules of acetyl-CoA for anabolism, mostly gluconeogenesis. TCA and glyoxylate cycles bifurcate at the level of isocitrate. In the TCA cycle, isocitrate is converted to α-ketoglutarate by isocitrate dehydrogenase, thus directing the carbon flux to the TCA cycle. In glyoxylate cycle, isocitrate is converted to glyoxylate and succinate, thus committing the carbon flux to anabolism. The dramatically increased mRNA levels of glyoxylate cycle genes *ICL1* and *MLS1*, encoding isocitrate lyase and malate synthase, respectively, and the significantly decreased mRNA level of the TCA cycle gene *IDH1*, encoding isocitrate dehydrogenase (Fig. 2), suggests that the carbon flux through TCA and glyoxylate cycles is significantly deregulated in *mec1*Δ*sml1*Δ cells. It is tempting to speculate that the decreased mRNA levels of glyoxylate cycle and gluconeogenesis genes in *rad53*Δ*sml1*Δ, *chk1*Δ, and *dun1*Δ cells are responsible for the defect in biosynthetic processes, required for passing the Start point of the cell cycle and entering S phase (Fig. 1*B*). In contrast, *mec1*Δ*sml1*Δ cells with elevated mRNA levels of glyoxylate cycle and gluconeogenesis genes appear to be able to pass the Start point (Fig. 1*B*).

### Transcription of RNR2, RNR3, and RNR4 is upregulated in dun1Δ cells

The increased mtDNA copy number in *dun1Δ* cells grown on acetate (Fig. 1*D*) indicates that the synthesis of dNTPs is upregulated upon *DUN1* inactivation. This is counterintuitive, since Dun1p regulates transcription of *RNR2*, *RNR3*, and *RNR4* genes by relieving repression mediated by Crt1p. Crt1p is a DNA binding protein that represses transcription by recruiting the general repressors Tup1p and Ssn6p. Following DNA damage or replication stress, Crt1p is hyperphosphorylated and dissociates from chromatin, leading to derepression (22, 36, 37). In the absence of genotoxic or replication stress, one of the main functions of Mec1p and Rad53p is to elevate dNTP synthesis during S phase of the cell cycle by relieving Crt1p-mediated repression of *RNR2-4* genes and by inactivating RNR inhibitor Sml1p. Correspondingly, *mec1Δ* and *rad53Δ* cells are viable only if harboring *crt1Δ* or *sml1Δ* mutations (22, 32).

**Figure 3:**
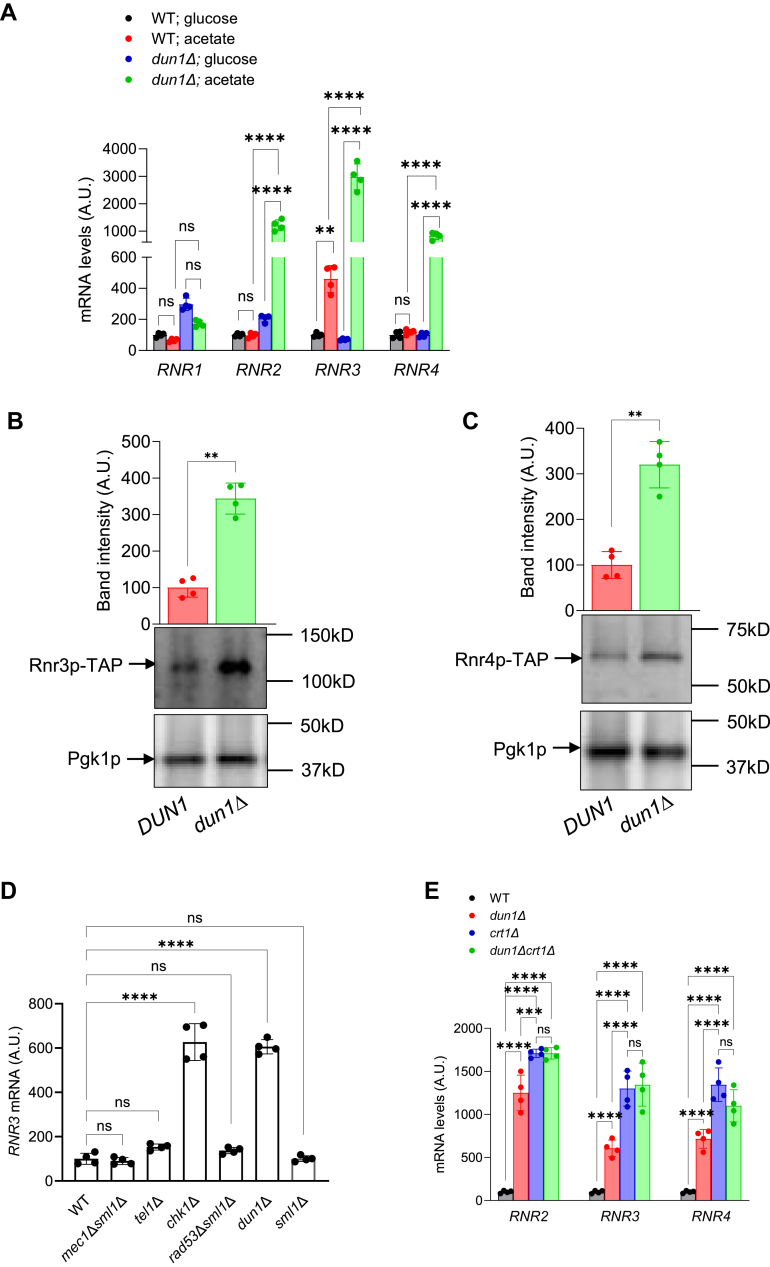
**Dun1p represses *RNR2*, *RNR3*, and *RNR4* genes by a Crt1p-dependent mechanism**. *A*, mRNA levels of *RNR1*, *RNR2*, *RNR3*, and *RNR4* in wildtype (WT) and *dun1*Δ cells grown in YPD medium (glucose) or YPA medium (acetate). *B*, *C*, Rnr3p-TAP and Rnr4p-TAP levels in *DUN1* and *dun1*Δ cells growing exponentially in YPA medium. Western blot was performed four times, and representative results are shown. The intensities of Rnr3p-TAP and Rnr4p-TAP bands were quantified by densitometry and normalized with Pgk1p as a loading control using NIH ImageJ software. Statistical significance was evaluated by two-tailed paired *t* test (∗*p* < 0.05, ∗∗*p* < 0.01, ∗∗∗*p* < 0.001). *D*, mRNA levels of *RNR3* in wildtype (WT), *mec1*Δ*sml1*Δ, *tel11*Δ, *chk1*Δ, *rad53*Δ*sml1*Δ, and *dun1*Δ cells grown in YPA medium. The data are means ± SD from four biologically independent experiments; ∗*p* < 0.05, ∗∗*p* < 0.01, ∗∗∗*p* < 0.001, ∗∗∗∗*p* < 0.0001 by one-way ANOVA and Dunnett's multiple comparisons test. *E*, mRNA levels of *RNR2*, *RNR3*, and *RNR4* in wildtype (WT), *dun1*Δ, *crt1*Δ, and *dun1*Δ*crt1*Δ cells grown in YPA medium. (*A*, *E*) The data are means ± SD from four biologically independent experiments; ∗∗*p* < 0.01, ∗∗∗*p* < 0.001, ∗∗∗∗*p* < 0.0001 by two-way ANOVA and Tukey's test. (*A*) The results are shown as arbitrary units (A.U.), and the mean for WT cells grown in glucose was set as 100 A U. (*B*, *C*, *D*, *E*) The results are shown as arbitrary units (A.U.), and the mean for WT cells was set as 100 A U.

To get insight into regulation of transcription of the *RNR2-4* genes in cells grown on acetate, we determined mRNA levels of *RNR2*, *RNR3*, and *RNR4* genes in WT and *dun1Δ* cells grown on glucose or acetate (Fig. 3*A*). mRNA level of *RNR1* was included for comparison; transcription of *RNR1* is regulated in a cell cycle–dependent manner by the transcription complex MBF and by high-mobility group domain protein Ixr1p, but not by Crt1p (38, 39, 40, 41). The results allow two important conclusions. First, in cells grown on acetate, the mRNA levels of *RNR2*, *RNR3*, and *RNR4* are significantly elevated in *dun1Δ* in comparison with WT cells (Fig. 3*A*). The increased mRNA levels of the *RNR* genes in *dun1Δ* cells grown on acetate are in agreement with elevated protein levels of Rnr3p-TAP (Fig. 3*B*) and Rnr4p-TAP (Fig. 3*C*). Second, the mRNA level of *RNR3*, unlike that of *RNR2* and *RNR4*, is upregulated in WT cells grown on acetate in comparison with WT cells grown on glucose. The fact that *RNR2*, *RNR3*, and *RNR4* are repressed by Crt1p, but only *RNR3* is induced by acetate indicates that the increase in *RNR3* mRNA level on acetate occurs by a mechanism independent of Crt1p and most likely independent of Dun1p. Interestingly, previous study found that increased abundance of Rnr3p in cells grown on nonfermentable carbon sources requires Mec1p (42).

The unexpected increase in *RNR3* mRNA level in *dun1Δ* cells on acetate prompted us to test whether inactivation of another checkpoint kinase also leads to derepression of *RNR3* (Fig. 3*B*). The results show that *CHK1* inactivation also causes derepression of *RNR3* (Fig. 3*D*). Interestingly, *RNR3* inactivation leads to growth defect on nonfermentable carbon sources and displays genetic interactions with *tom6Δ* mutation; *TOM6* encodes a component of the mitochondrial TOM (translocase of outer membrane) complex, responsible for mitochondrial protein import. These findings implicate *RNR3* and dNTP synthesis in mitochondrial function (42).

To determine whether *dun1Δ* mutation affects expression of *RNR2*, *RNR3*, and *RNR4* by relieving the Crt1p-mediated repression or independently of Crt1p, we compared mRNA levels of *RNR2*, *RNR3*, and *RNR4* in *dun1Δ*, *crt1Δ*, and *dun1Δcrt1Δ* cells (Fig. 3*E*). Since the effect of *dun1Δ* and *crt1Δ* mutations on the mRNA levels is not additive for any of the *RNR2-4* genes, we conclude that in cells grown on acetate, Dun1p regulates transcription of the *RNR2-4* genes by maintaining the Crt1p-mediated repression.

### Dun1p is required for recruitment of Crt1p to RNR promoters

**Figure 4:**
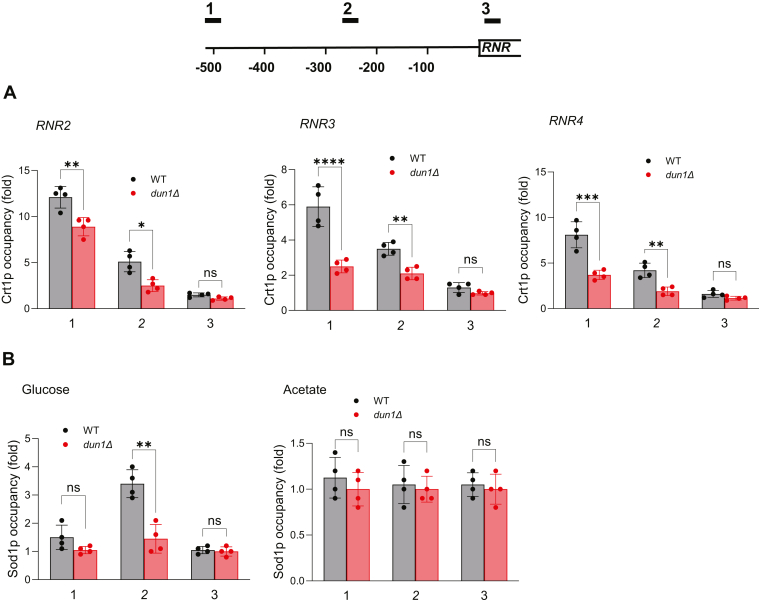
**Dun1p is required for recruitment of Crt1p to the promoters of *RNR* genes**. *A*, occupancy of Crt1p at the indicated positions of promoters and coding regions of *RNR2*, *RNR3*, and *RNR4* in the wildtype (WT) and *dun1*Δ cells grown in YPA medium. *B*, occupancy of Sod1p at the indicated positions within the promoter and coding region of *RNR3* gene in the wildtype (WT) and *dun1*Δ cells grown in YPD medium (glucose) and treated with 0.4 mM hydrogen peroxide for 20 min and grown in YPA medium (acetate). (*A*, *B*) The data are means ± SD from four biologically independent experiments; ∗*p* < 0.05, ∗∗*p* < 0.01, ∗∗∗*p* < 0.001, ∗∗∗∗*p* < 0.0001 by two-way ANOVA and Tukey’s test. RNR, ribonucleotide reductase.

To determine whether Dun1p regulates transcription of the *RNR2-4* genes directly by affecting the occupancy of Crt1p at the corresponding promoters, we performed a chromatin immunoprecipitation experiment to determine occupancy of myc-tagged Crt1p within 500 kb upstream of *RNR2*, *RNR3*, and *RNR4* coding regions (Fig. 4*A*). As expected, Crt1p was recruited to the corresponding promoters; the maximum occupancy was found around 500 bp upstream of the translational start sites. Importantly, the Crt1p occupancies at all three promoters were significantly decreased in *dun1Δ* cells in comparison with the WT cells (Fig. 4*A*). In addition to Crt1p, transcription of the *RNR3* gene is regulated by superoxide dismutase Sod1p (43). By dismutating superoxide (O_2_^-^) into oxygen and hydrogen peroxide, Sod1p is part of anti-oxidative system that protects cells from oxidative damage. Surprisingly, Sod1p was found to translocate to the nucleus in response to elevated reactive oxygen species. This translocation requires Mec1p and Dun1p. Dun1p binds and phosphorylates Sod1p. Dun1p-phosphorylated Sod1p then binds to promoters and activates transcription of oxidative resistance and repair genes, including *RNR3* (43). In addition, *SOD1* inactivation reduces hydroxyurea (HU)-triggered induction of *RNR3* (44). We detected recruitment of Sod1p to the *RNR3* promoter in glucose-grown cells treated with hydrogen peroxide (Fig. 4*B*). The maximum occupancy was found around 250 bp upstream of the *RNR3* translational start site, indicating that the recruitment is independent of Crt1p. The occupancy was significantly reduced in *dun1Δ* cells. Importantly, despite significant effort, we were not able to detect Sod1p recruitment to the *RNR3* promoter in acetate-grown cells (Fig. 4*B*). We conclude that Sod1p is not recruited to the *RNR3* promoter in acetate-grown cells and thus does not play a role in increased transcription of the *RNR2-4* genes in *dun1Δ* cells.

### Kinase activity of Dun1p and signaling from Rad53p are not required for repression of RNR2-4 genes and recruitment of Crt1p to RNR promoters

To examine whether the protein kinase activity of Dun1p and/or phosphorylation of Dun1p by Rad53p are required for repression of *RNR2-4* genes, we transformed *dun1*Δ cells separately with (i) empty low-copy centromeric plasmid pRS416, (ii) pRS416 plasmid expressing WT *DUN1* under the control of its own promoter, (iii) pRS416 plasmid expressing *dun1-D328 A* allele, and (iv) pRS416 plasmid expressing *dun1-T380 A* allele. Asp-328 residue is in the protein kinase catalytic site of Dun1p and its substitution with Ala (D328 A) completely abolishes Dun1p protein kinase activity (45, 46, 47). Thr-380 residue is in the activation loop of Dun1p, and when the checkpoint kinase cascade is activated, Rad53p activates Dun1p by phosphorylating Thr-380. *dun1-T380 A* mutants are not able to phosphorylate downstream targets, such as Sml1p (47).

**Figure 5:**
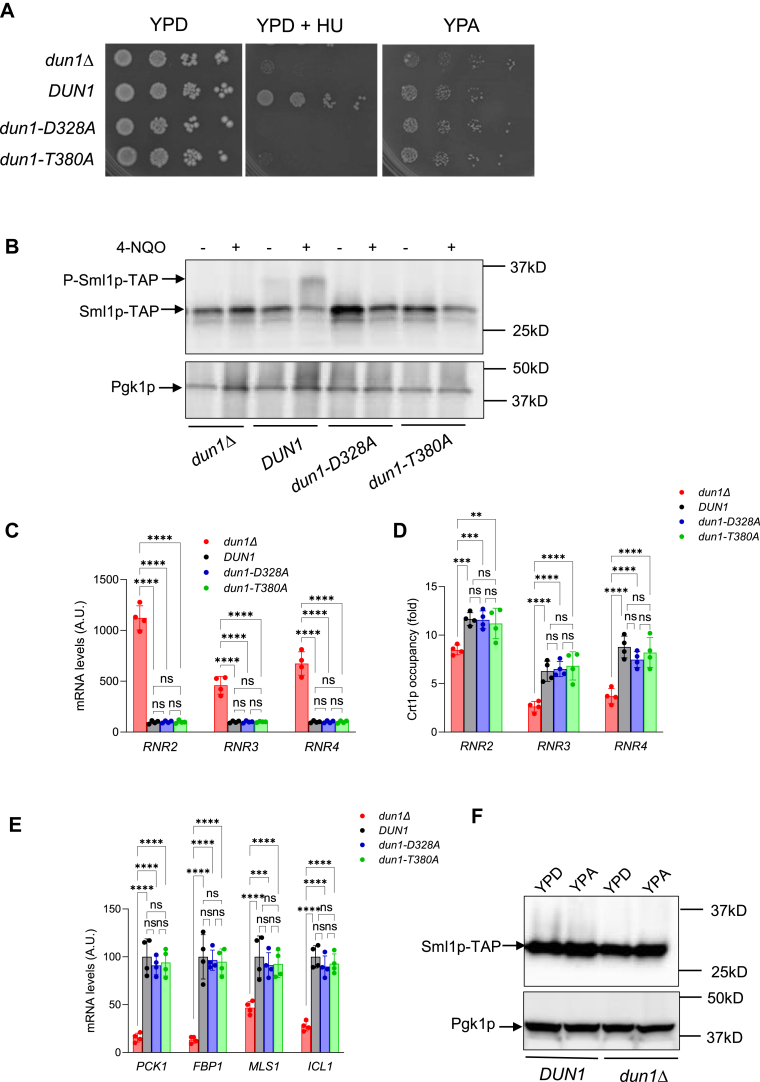
**Kinase activity of Dun1p and signaling from Rad53p are not required for repression of RNR2-4 genes and recruitment of Crt1p to RNR promoters**. *A*, *dun1*Δ, *dun1-D328 A*, and *dun1-T380 A* cells are not able to grow in the presence of 50 mM hydroxyurea (HU). 10-fold serial dilutions of *dun1*Δ cells transformed with pRS416 empty plasmid (RM245), pRS416-*DUN1* (RM233), pRS416-*dun1-D328 A* (RM237), or pRS416-*dun1-T380 A* (RM241) were spotted onto YPD plates, YPD plates containing 50 mM HU, and YPA plates and grown for 48 h, 72 h, and 96 h, respectively. *B*, Sml1p-TAP levels in *dun1*Δ *SML1-TAP* cells transformed with pRS416 empty plasmid (RM249), pRS416-*DUN1* (RM251), pRS416-*dun1-D328 A* (RM253), or pRS416-*dun1-T380 A* (RM255). Cells growing exponentially in YPD were or were not treated with 10 μg/ml 4-NQO for 1 h. Western blot was performed three times, and representative results are shown. *C*, *E*, mRNA levels of *RNR2*, *RNR3*, and *RNR4* (*C*) and mRNA levels of *PCK1*, *FBP1*, *MLS1*, *and ICL1* (*E*) in *dun1*Δ cells transformed with pRS416 empty plasmid (RM245), pRS416-*DUN1* (RM233), pRS416-*dun1-D328 A* (RM237), or pRS416-*dun1-T380 A* (RM241). *D*, occupancy of Crt1p at position 1 (see Fig. 4) of *RNR2*, *RNR3*, and *RNR4* promoters in *dun1*Δ cells transformed with empty plasmid (RM257), pRS416-*DUN1* (RM259), pRS416-*dun1-D328 A* (RM261), or pRS416-*dun1-T380 A* (RM263). *C*–*E*, precultures were grown in SC-Ura medium to maintain the plasmids, and subsequently grown in YPD or YPA medium for two generations. *C*, *D*, *E*, the data are means ± SD from four biologically independent experiments; ∗*p* < 0.05, ∗∗*p* < 0.01, ∗∗∗*p* < 0.001, ∗∗∗∗*p* < 0.0001 by two-way ANOVA and Tukey's test. *C*, *E*, the results are shown as arbitrary units (A.U.), and the mean for *DUN1* cells was set as 100 A U. *F*, Sml1p-TAP levels in *DUN1SML1-TAP* and *dun1*Δ *SML1-TAP* cells growing exponentially in YPD or YPA. Western blot was performed three times, and representative results are shown. RNR, ribonucleotide reductase.

To confirm the phenotypes of *dun1*Δ, *DUN1*, *dun1-D328 A*, and *dun1-T380 A* cells, we tested their ability to grow in the presence of the RNR inhibitor HU and their ability to phosphorylate Dun1p′s downstream target Sml1p. As expected, only *DUN1*, but not *dun1*Δ, *dun1-D328 A*, or *dun1-T380 A* cells were able to grow in the presence of 50 mM HU (Fig. 5*A*). Rather surprisingly, not only *DUN1* but also *dun1-D328 A* and *dun1-T380 A* cells grew slightly faster than *dun1*Δ cells on YPA (Fig. 5*A*). When exposed to genotoxic chemical 4-nitroquinoline 1-oxide, only *DUN1* cells were able to phosphorylate Sml1p (Fig. 5*B*). These results suggest that the catalytic activity of Dun1p or Rad53p-mediated signaling to Dun1p are not required for normal growth on acetate. Similarly, repression of *RNR2-4* genes (Fig. 5*C*), Crt1p occupancy at *RNR2-4* promoters (Fig. 5*D*), and transcription of gluconeogenetic and glyoxylate cycle genes (Fig. 5*E*) do not require catalytic activity of Dun1p or Rad53p-mediated signaling to Dun1p (Fig. 5, *C* and *D*). These data are consistent with the possibility that Dun1p contributes to transcriptional regulation independently of its kinase activity as a structural component by binding to Crt1p or other proteins involved in gene regulation (Fig. 5, *A*–*F*). Noncatalytic mechanism of transcriptional regulation was described for yeast mitogen-activated protein kinase Mpk1p (48).

## Discussion

The key finding of this study is that checkpoint kinases are required for normal growth on nonfermentable carbon source acetate and that inactivation of individual kinases results in distinct transcription changes of metabolic genes in the absence of genotoxic stress. Perhaps the most surprising was the finding that on acetate media, Dun1p functions independently of its kinase activity and signaling from Rad53p to repress transcription of *RNR2*, *RNR3*, and *RNR4* genes by maintaining Crt1p-mediated repression. The elevated transcription of the *RNR2-4* genes triggered by Dun1p deletion is likely associated with increased activity of the RNR enzyme and increased dNTP levels, since it results in significantly upregulated mtDNA copy number (Fig. 1*D*); cellular dNTP levels parallel mtDNA copy number (24, 31).

It is well established that DDR and checkpoint kinases respond to genotoxic stress by regulating major metabolic pathways, including glycolysis, TCA cycle, and PPP (49, 50, 51, 52, 53). The purpose of these DDR-induced metabolic changes is to prevent further DNA damage, provide metabolites required for DNA repair, including dNTPs, and minimize damage caused by reactive oxygen species. Conversely, defect in mitochondrial Fe-S cluster biogenesis activates DDR (54). In mammalian cells, ATM activates PPP to increase NADPH, an important antioxidant, and nucleotide synthesis (55). In yeast, HU-induced replication stress leads to PPP activation by a mechanism that involves yeast AMPK ortholog Snf1p. Surprisingly, NADPH produced by the PPP facilitates recruitment of replication protein A to single stranded DNA, enhancing Mec1p activation (56). We found that in yeast cells grown on glucose, DDR activates respiration to elevate synthesis of dNTPs, required for DNA repair (30). The underlying mechanism involves downregulation of histone transcription, leading to altered chromatin structure and increased transcription of TCA cycle, ETC, and oxidative phosphorylation genes (30, 57).

Surprisingly, our data indicate that checkpoint kinases are involved in transcriptional regulation of metabolism in the absence of genotoxic stress. Are checkpoint kinases activated on nonfermentable carbon source in the absence of genotoxic stress? And if they are, what is the mechanism? Typically, checkpoint kinases are activated in response to DNA damage, replication stress, and during normal uninterrupted S phase (7). The canonical pathway for activation of checkpoint kinases depends on the recruitment of checkpoint kinases to single-stranded DNA or DNA double-strand breaks. Mec1p is activated during normal undisturbed S phase, during replication stress, or after DNA damage by being recruited to single-stranded DNA coated by the replication protein A complex. Recruitment and activation of Mec1p also requires Ddc2p, 9-1-1 complex (Ddc1p, Mec3p, and Rad17p), Dpb11p, and Dna2p (7). Tel1p is recruited and activated at DNA double-strand breaks by the MRX complex (Mre11, Rad50p, and Xrs2p). In addition to this canonical pathway, ATM in human cells can be activated in the absence of DNA damage by oxidative stress (58, 59) by a mechanism that depends on ATM dimerization through oxidation of Cys residues and formation of disulfide crosslinks. Since this Cys residue is not conserved in lower eukaryotes, including yeast *S*. *cerevisiae*, yeast Tel1p cannot be activated by this mechanism. However, we cannot exclude the possibility that oxidative stress in yeast cells activates other checkpoint kinases by a mechanism reminiscent of mammalian ATM. An alternative mechanism for activation of checkpoint kinases was described by a recent study that employed systematic analysis of protein localization changes caused by replication stress (60). This work identified a novel noncanonical pathway of Mec1p- and Tel1p-independent Rad53p activation that depends on transcription factor Rtg3p, a component of the RTG signaling pathway. The authors proposed a model in which replication stress results in Mec1p- and Tel1p-independent nuclear import of Rtg3p, leading to transcription induction of Rtg3p target genes. Rtg3p target gene(s) then promote Rad53p phosphorylation and activation (60). The possible involvement of the RTG pathway in DDR signaling is also indicated by the finding that inactivation of *MEC1*, *RAD53*, or *RTG1*, encoding another component of the RTG pathway, leads to sensitivity to glutamine analog 6-diazo-5-oxo-L-norleucine (61). The RTG signaling pathway monitors mitochondrial function and transmits signals to the nucleus. RTG signaling is triggered by mitochondrial dysfunction; however, it is also required for the activation of mitochondrial metabolism when cells are grown in nonfermentable carbon sources (62). The key event in the RTG pathway is the translocation of the Rtg1/3p transcription factor from the cytoplasm to the nucleus, with a concomitant activation of the RTG target genes (63). Upregulation of peroxisomal citrate synthase, encoded by the *CIT2* gene, is a hallmark of RTG signaling activation (64). It is possible that growth on acetate that triggers RTG signaling and nuclear translocation of the Rtg1/Rtg3p transcription factor also promotes Rtg3p-mediated activation of Rad53p by the noncanonical pathway of Mec1p- and Tel1p-independent Rad53p activation (60).

However, we believe that it is more likely that checkpoint kinase(s) are not activated by growth in nonfermentable carbon sources and that they function in their nonactivated state or that only the partial periodic activation during each S phase is sufficient for normal growth on nonfermentable carbon sources (Fig. 5). Examples of genotoxic stress-independent role of checkpoint kinases include formation of Snf1p-Mec1p-Atg1p complex on mitochondria that is essential for glucose starvation–induced autophagy (65) and the role of Rad53p in regulation of histone levels (66, 67). Our results provide another example for a genotoxic stress-independent role of checkpoint kinases. Dun1p represses transcription of *RNR2-4* genes by promoting Crt1p occupancy in the *RNR2-4* promoters independently of its kinase activity and signaling from Rad53p (Fig. 5, *C* and *D*). Perhaps the underlying mechanism involves interaction between Dun1p and Crt1p proteins that promotes Crt1p occupancy at the *RNR2-4* promoters. Many protein kinases, including checkpoint kinases, associate with genes and regulate their transcription (68, 69).

One of the major metabolic roles of PPP is synthesis of ribose 5-phosphate, precursor for both ribonucleotide triphosphates (NTPs) and dNTPs. The cellular levels and balance between dNTPs and NTPs are important for normal cell physiology, including DNA and RNA synthesis. Since Dun1p appears to be required for carbon source–mediated control of *RNR2-4* transcription, it is possible that Dun1p also contributes to the regulation of dNTPs synthesis and dNTPs/NTPs ratio according to different nutritional conditions, growth rate, and metabolic flux through PPP (23, 25, 26).

Rad53p, but not the upstream kinases Mec1p or Tel1p, prevents accumulation of excess histones, arguing that the basal kinase activity of Rad53p is sufficient to trigger phosphorylation and degradation of nonchromatinized histones (66, 67). In the absence of genotoxic stress, Rad53p also regulates transcription of histone genes by phosphorylating histone genes-specific transcription factor Spt21p (70). Correspondingly, inactivation of Rad53p results in toxic accumulation of histones when cells are grown in media with limiting concentration of glucose. This role is specific to Rad53p and is not shared by other checkpoint kinases (70).

Proliferating cells need to maintain a delicate balance between histone and DNA synthesis to ensure correct stoichiometric amounts for chromatin assembly and to avoid genome instability (71, 72). Rad53p has an important role in maintaining this balance (66, 67, 68, 69, 70) by preventing accumulation of free nonchromatinized histones that are toxic to the cell (8, 73, 74). What is the significance of Dun1p-mediated repression of the *RNR2-4* genes on acetate medium? Since yeast cells grow significantly slower on acetate than on glucose, the Dun1p-mediated repression of the *RNR* genes might be important for adjusting dNTP synthesis to lower demand due to a slower rate of DNA replication in order to balance dNTP synthesis and DNA replication with histone synthesis. Deregulated synthesis and accumulation of dNTPs results in cell cycle arrest (75).

Overall, our data suggest that when cells are grown on suboptimal carbon source in the absence of genotoxic stress, Dun1p-mediated repression of *RNR2*, *RNR3*, and *RNR4* genes provides additional mechanism for coordinating metabolism with synthesis of dNTPs, DNA, and histones. We speculate that the role of Dun1p in the regulation of dNTP synthesis complements the role of Rad53p in histone synthesis and degradation and represents another tool in the repertoire of checkpoint kinases for balanced synthesis of chromatin.

## Experimental procedures

### Yeast strains, plasmids, and media

All yeast strains are listed in Table 1. Standard genetic techniques were used to manipulate yeast strains and to introduce mutations from non-W303 strains into the W303 background (76, 77). DNA fragments, including coding region, 500 bp of upstream sequence, and 300 bp downstream sequence for *DUN1*, *DUN1-D328 A*, and *DUN1-T380 A*, were synthesized, inserted in pRS416 vector, and sequenced by Twist Biosciences. Cells were grown in YEP medium (1% yeast extract, 2% Bacto peptone), containing 2% glucose (YPD) or 2% acetate (YPA) or under selection in synthetic complete medium containing 2% glucose and, when appropriate, lacking specific nutrients to select for a strain with a particular genotype.

**Table 1:** Yeast strains used in this study

Strain	Genotype	Source/Ref.
W303-1a	*MATa ade2-1 his3-11*,*15 leu2-3112 trp1-1 ura3-1 ssd1-d2 can1-100*	R. Rothstein
W303-1α	*MAT*α *ade2-1 his3-11*,*15 leu2-3112 trp1-1 ura3-1 ssd1-d2 can1-100*	R. Rothstein
W303	*MATa/MAT*α *ade2-1/ade2-1 his3-11*,*15/his3-11*,*15 leu2-3112/leu2-3112trp1-1/trp1-1ura3-1/ura3-1 can1-100/can1-100*	R. Rothstein
SN141	*MATa ade2-1 his3-11*,*15 leu2-3112 trp1-1 ura3-1 ssd1-d2 can1-100* *dun1*:*:HIS3*	(70)
SN159	*MATa ade2-1 his3-11*,*15 leu2-3112 trp1-1 ura3-1 ssd1-d2 can1-100* *tel1*:*:HIS3*	(30)
SN136	*MATα ade2-1 his3-11*,*15 leu2-3112 trp1-1 ura3-1 ssd1-d2 can1-100* *chk1*:*:HIS3*	(30)
SN117	*MATa ade2-1 his3-11*,*15 leu2-3112 trp1-1 ura3-1 ssd1-d2 can1-100* *mec1*:*:HIS3 sml1*:*:KAN*	(30)
LG606	*MATa ade2-1 his3-11*,*15 leu2-3112 trp1-1 ura3-1 ssd1-d2 can1-100* *rad53*:*:KAN sml1*:*:HYG*	(30)
LG603	*MATa ade2-1 his3-11*,*15 leu2-3112 trp1-1 ura3-1 ssd1-d2 can1-100* *sml1*:*:HYG*	(30)
SZy1051	*MATa hisΔ1 leu2Δ0 met15Δ0 ura3Δ0 sod1Δ*:*:KanMX* *pRS415 (SOD1-MYC9)*	(43)
SN711	*MATa ade2-1 his3-11*,*15 leu2-3112 trp1-1 ura3-1 ssd1-d2 can1-100* *sod1Δ*:*:KanMX pRS415 (SOD1-MYC9)*	This study
SN713	*MATa ade2-1 his3-11*,*15 leu2-3112 trp1-1 ura3-1 ssd1-d2 can1-100* *dun1*:*:HIS3 sod1*:*:KanMX pRS415 (SOD1-MYC9)*	This study
Y588	*MATa can1-100 ade2-1 his3-11*,*15 leu2-3112 trp1-1 ura3-1* *crt1-Δ1*:*:LEU2*:*:CRT1-12xMYC-HIS3*	(22)
SN642	*MATa ade2-1 his3-11*,*15 leu2-3112 trp1-1 ura3-1 ssd1-d2 can1-100* *crt1-Δ1*:*:LEU2*:*:CRT1-12xMYC-HIS3*	This study
SN645	*MATa ade2-1 his3-11*,*15 leu2-3112 trp1-1 ura3-1 ssd1-d2 can1-100* *dun1*:*: KanMX crt1-Δ1*:*:LEU2*:*:CRT1-12xMYC-HIS3*	This study
RM245	*MATa ade2-1 his3-11*,*15 leu2-3112 trp1-1 ura3-1 ssd1-d2 can1-100* *dun1*:*:HIS3 [pRS416]*	This study
RM233	*MATa ade2-1 his3-11*,*15 leu2-3112 trp1-1 ura3-1 ssd1-d2 can1-100* *dun1*:*:HIS3 [pRS416-DUN1]*	This study
RM237	*MATa ade2-1 his3-11*,*15 leu2-3112 trp1-1 ura3-1 ssd1-d2 can1-100* *dun1*:*:HIS3 [pRS416-dun1-D328 A]*	This study
RM241	*MATa ade2-1 his3-11*,*15 leu2-3112 trp1-1 ura3-1 ssd1-d2 can1-100* *dun1*:*:HIS3 [pRS416-dun1-T380 A]*	This study
RM249	*MATa ade2-1 his3-11*,*15 leu2-3112 trp1-1 ura3-1 ssd1-d2 can1-100* *dun1*:*: KanMX SML1-TAP*:*:HIS3 [pRS416]*	This study
RM251	*MATa ade2-1 his3-11*,*15 leu2-3112 trp1-1 ura3-1 ssd1-d2 can1-100* *dun1*:*: KanMX SML1-TAP*:*:HIS3 [pRS416-DUN1]*	This study
RM253	*MATa ade2-1 his3-11*,*15 leu2-3112 trp1-1 ura3-1 ssd1-d2 can1-100* *dun1*:*: KanMX SML1-TAP*:*:HIS3 [pRS416-dun1-D328 A]*	This study
RM255	*MATa ade2-1 his3-11*,*15 leu2-3112 trp1-1 ura3-1 ssd1-d2 can1-100* *dun1*:*: KanMX SML1-TAP*:*:HIS3 [pRS416-dun1-T380 A]*	This study
RM257	*MATa ade2-1 his3-11*,*15 leu2-3112 trp1-1 ura3-1 ssd1-d2 can1-100* *dun1*:*:KanMX crt1-Δ1*:*:LEU2*:*:CRT1-12xMYC-HIS3 [pRS416]*	This study
RM259	*MATa ade2-1 his3-11*,*15 leu2-3112 trp1-1 ura3-1 ssd1-d2 can1-100* *dun1*:*:KanMX crt1-Δ1*:*:LEU2*:*:CRT1-12xMYC-HIS3 [pRS416-DUN1]*	This study
RM261	*MATa ade2-1 his3-11*,*15 leu2-3112 trp1-1 ura3-1 ssd1-d2 can1-100* *dun1*:*:KanMX crt1-Δ1*:*:LEU2*:*:CRT1-12xMYC-HIS3 [pRS416-dun1-D328 A]*	This study
RM263	*MATa ade2-1 his3-11*,*15 leu2-3112 trp1-1 ura3-1 ssd1-d2 can1-100* *dun1*:*:KanMX crt1-Δ1*:*:LEU2*:*:CRT1-12xMYC-HIS3 [pRS416-dun1-T380 A]*	This study
RM225	*MATa ade2-1 his3-11*,*15 leu2-3112 trp1-1 ura3-1 ssd1-d2 can1-100* *RNR3-TAP*:*:HIS3*	This study
RM226	*MATa ade2-1 his3-11*,*15 leu2-3112 trp1-1 ura3-1 ssd1-d2 can1-100* *dun1*:*:KanMX RNR3-TAP*:*:HIS3*	This study
RM229	*MATa ade2-1 his3-11*,*15 leu2-3112 trp1-1 ura3-1 ssd1-d2 can1-100* *RNR4-TAP*:*:HIS3*	This study
RM230	*MATa ade2-1 his3-11*,*15 leu2-3112 trp1-1 ura3-1 ssd1-d2 can1-100* *dun1*:*:KanMX RNR4-TAP*:*:HIS3*	This study

### Real-time RT-qPCR

The procedures to extract total RNA from yeast cells and perform real-time reverse transcription quantitative PCR were as previously described (30, 78). The primers used are as follows:

*RDN25* (5′-GGAATGTAGCTTGCCTCGGT-3′ and 5′-TTACGTCGCAGTCCTCAGTC-3′), *RNR1* (5′- ACCGCTCGTATATCACGCTTAT-3′ and 5′- TTGTGACACCTTCATAGACA CCA-3′), *RNR2* (5′-CGTGCCGAAGCTTCTTTCTG-3′ and 5′-GTCAGAAGCGGCGA AAAAGG-3′), *RNR3* (5′- TATTAAAAGAGACGGCCGCA-3′ and 5′- AGCATCAATACG GTTTGGGTC-3′), *RNR4* (5′- TTCGTGATGTTCCCTATCAAA-3′ and 5′- GTGTCCTTAGCCAAT TCGATT-3′), *CIT1* (5′- CAGCGATATTATCAACAACTAGCA-3′ and 5′- TAGTGGCGAG CATTCAATAGTG-3′), *ACO1* (5′- TGTTCGTGGTCTTGCGACA-3′ and 5′- CGTTTCCACA TTCTGCTTGTAGT-3′), *IDH1* (5′-TGCTTAACAGAACAATTGCTAAGAG-3′ and 5′-AAC ACCGTCACCAGGTATCAA-3′), *COX1* (5′-CAACAAATGCAAAAGATATTG CAG-3′ and 5′-AATATTGTGAACCAGGTGCAGC-3′), *FBP1* (5′-TCTCACACCATCAGACGTGC-3′ and 5′-CCACTAGCCCTCATGGCATT-3′), *PYC1* (5′- TCGCCGGCTTGAGAGATAAC-3′ and 5′- TCAGCCTTTTGTTTGTGCGT-3′), *PCK1* (5′- AGCGGTGCATTGATCGCTTA-3′ and 5′- TTTATTGACCGGACCCCACC-3′), *MLS1* (5′- TGGTGGATGTTGATAAGGAGCC-3′ and 5′- AGGAGCCCGAGTCTAATTTCT-3′), *ICL1* (5′-CTAGATGCAGATGCTGCCGA-3′ and 5′-GGAATGTCCCGCGTCTAACA-3′), *ZWF1* (5′-TCCCGCCTTATTTGGGCTTT-3′ and 5′-TAGGACACGGGACTTCAGGT-3′), and *CIT2* (5′- AGAGATTT AGCGAAATCTACCCC-3′ and 5′- CCTCTCATACCACCATATACCTGTT-3′).

### Chromatin immunoprecipitation assays

*In vivo* chromatin crosslinking and immunoprecipitation was performed essentially as described (78). Immunoprecipitation was performed with anti-myc monoclonal antibody (9B11; 2276S; Cell Signaling). The primers used for real-time PCR are as follows: *POL1* (5′-TCCTGACAAAGA AGGCAATAGAAG-3′ and 5′-TAAAACACCCTGATCCACCTCTG-3′), *RNR2-3* (5′-CGTGCCGAAGCTTCTTTCTG-3′ and 5′-GTCAGAAGCGGCGAAAAAGG-3′), *RNR2-1* (5′-GGTAGTGTTTGCGCGTTACC-3′ and 5′-CAGCTTTGGAAGGGGTCTCT-3′), *RNR2-2* (5′-AGCAAAGCAAAGGAGGGGAA-3′ and 5′-GATGGTAACGCGCAAAC ACT-3′), *RNR3-3* (5′- TATTAAAAGAGACGGCCGCA-3′ and 5′- AGCATCAATACGGTTTGGGTC-3′), *RNR3-1* (5′-TGCTGACGCGTTTCGTTTTT-3′ and 5′-TGCTGCTGCTATTCTTGCTTG-3′), *RNR3-2* (5-CTCAAAGGGGCAAAAACCCG-3′ and 5′-AAAAACGAAACGCGTCA GCA-3′), *RNR4-3* (5′- TTCGTGATGTTCCCTATCAAA-3′ and 5′- GTGTCCTTAGCCAATTCGATT-3′), *RNR4-1* (5′-CCACCTCTGTCCCCTTACACT-3′ and 5′-TAATTGTGTGCGTTGCG CGG-3′), and *RNR4-2* (5′-GGTTTCTGACCCCACCTCTG-3′ and 5′-GGGGTTGTGTGGCTC AAAAAG-3′).

### Western blotting

Western blotting was performed as described, and samples of whole cell extracts corresponding to 25 μg of proteins were analyzed in each lane (24). Membranes were probed with anti-TAP tag polyclonal antibody (CAB1001, Thermo Fisher Scientific) at a dilution of 1:1000 and anti-Pgk1p monoclonal antibody (22C5D8; 459,250; Invitrogen) at a dilution of 1:3000 (24). Signal was detected with ECL prime Western blotting detection reagent (RPN2236, Amersham) using Bio-Rad ChemiDoc imaging system.

### Flow cytometry

The DNA contents of cells were determined by flow cytometry of Sytox Green stained cells using Sigma Millipore Guava easyCyte flow cytometer as described (79).

### mtDNA isolation and quantification

mtDNA isolation and quantification was performed as described (80). Cells were grown to an *A*_*600nm*_ of 0.6 in YPD or YPA medium. Cells were harvested by centrifugation and lysed in buffer containing 2% Triton X-100, 1% SDS, 100 mM NaCl, 10 mM Tris-HCl pH 8.0, and 1 mM EDTA with prechilled glass beads. The lysate was extracted with phenol and chloroform. RNA was digested with RNase, and total DNA was purified by phenol and chloroform. Relative mtDNA was quantified by real-time PCR using primers for *COX1*, and the results were normalized with primers for *ACT1*.

### Oxygen consumption

Oxygen consumption was measured as described (80, 81). Briefly, cells were grown to an *A*_600nm_ of 0.6 in YPD or YPA medium, and 9 × 10^6^ cells were harvested by centrifugation. Cells were resuspended in a buffer containing 10 mM Hepes, 25 mM K_2_HPO_4_, pH 7.0 and incubated at 30^°^C in an oxygen consumption chamber (Instech Laboratories, Inc.) connected to a NeoFOX fluorescence-sensing detector using NeoFOX software (Ocean Optics, Inc.). Results were calculated as pmol O_2_/10^6^ cell/sec and expressed as percentages of the WT value. The oxygen consumption rate in WT cells grown in YPD medium was 5.08 pmol/10^6^ cells/second and was set as 100%.

### Statistical analysis

All statistical analyses (one-way Anova, two-way Anova, paired *t* test, and tests for normal distribution of data and statistical outliers) were performed in GraphPad Prism 10.4.1 package.

## Data availability

All data are contained within the manuscript.

## Conflict of interest

The authors declare that they have no conflict of interest with the contents of this article.

## References

[1] Longhese M.P., Foiani M., Muzi-Falconi M., Lucchini G., Plevani P. (1998). DNA damage checkpoint in budding yeast. EMBO J..

[2] Kolodner R.D., Putnam C.D., Myung K. (2002). Maintenance of genome stability in *Saccharomyces cerevisiae*. Science.

[3] Putnam C.D., Jaehnig E.J., Kolodner R.D. (2009). Perspectives on the DNA damage and replication checkpoint responses in Saccharomyces cerevisiae. DNA Repair.

[4] Ciccia A., Elledge S.J. (2010). The DNA damage response: making it safe to play with knives. Mol. Cell.

[5] Putnam C.D., Kolodner R.D. (2017). Pathways and mechanisms that prevent genome instability in *Saccharomyces cerevisiae*. Genetics.

[6] Bell S.P., Labib K. (2016). Chromosome duplication in Saccharomyces cerevisiae. Genetics.

[7] Pardo B., Crabbé L., Pasero P. (2017). Signaling pathways of replication stress in yeast. FEMS Yeast Res..

[8] Gasch A.P., Huang M., Metzner S., Botstein D., Elledge S.J., Brown P.O. (2001). Genomic expression responses to DNA-damaging agents and the regulatory role of the yeast ATR homolog Mec1p. Mol. Biol. Cell.

[9] Jaehnig E.J., Kuo D., Hombauer H., Ideker T.G., Kolodner R.D. (2013). Checkpoint kinases regulate a global network of transcription factors in response to DNA damage. Cell Rep.

[10] Pizzul P., Casari E., Gnugnoli M., Rinaldi C., Corallo F., Longhese M.P. (2022). The DNA damage checkpoint: a tale from budding yeast. Front. Genet..

[11] Branzei D., Foiani M. (2006). The Rad53 signal transduction pathway: replication fork stabilization, DNA repair, and adaptation. Exp. Cell Res..

[12] Zhou C., Elia A.E., Naylor M.L., Dephoure N., Ballif B.A., Goel G. (2016). Profiling DNA damage-induced phosphorylation in budding yeast reveals diverse signaling networks. Proc. Natl. Acad. Sci. U S A..

[13] Pellicioli A., Foiani M. (2005). Signal transduction: how Rad53 kinase is activated. Curr. Biol..

[14] Chabes A., Georgieva B., Domkin V., Zhao X., Rothstein R., Thelander L. (2003). Survival of DNA damage in yeast directly depends on increased dNTP levels allowed by relaxed feedback inhibition of ribonucleotide reductase. Cell.

[15] Sabouri N., Viberg J., Goyal D.K., Johansson E., Chabes A. (2008). Evidence for lesion bypass by yeast replicative DNA polymerases during DNA damage. Nucleic Acids Res..

[16] Nordlund P., Reichard P. (2006). Ribonucleotide reductases. Annu. Rev. Biochem..

[17] Hofer A., Crona M., Logan D.T., Sjoberg B.-M. (2012). DNA building blocks: keeping control of manufacture. Crit. Rev. Biochem. Mol. Biol..

[18] Sanvisens N., de Llanos R., Puig S. (2013). Function and regulation of yeast ribonucleotide reductase: cell cycle, genotoxic stress, and iron bioavailability. Biomed. J..

[19] Mathews C.K. (2015). Deoxyribonucleotide metabolism, mutagenesis and cancer. Nat. Rev. Cancer.

[20] Ruskoski T.B., Boal A.K. (2021). The periodic table of ribonucleotide reductases. J. Biol. Chem..

[21] Zhao X., Chabes A., Domkin V., Thelander L., Rothstein R. (2001). The ribonucleotide reductase inhibitor Sml1 is a new target of the Mec1/Rad53 kinase cascade during growth and in response to DNA damage. EMBO J..

[22] Huang M., Zhou Z., Elledge S.J. (1998). The DNA replication and damage checkpoint pathways induce transcription by inhibition of the Crt1 repressor. Cell.

[23] Turcotte B., Liang X.B., Robert F., Soontorngun N. (2009). Transcriptional regulation of nonfermentable carbon utilization in budding yeast. FEMS Yeast Res..

[24] Nagar S., Mehta R., Kaur P., Liliah R.T., Vancura A. (2023). Tolerance to replication stress requires Dun1p kinase and activation of the electron transport chain. Biochim. Biophys. Acta Mol. Cell Res..

[25] Broach J.R. (2012). Nutritional control of growth and development in yeast. Genetics.

[26] Zaman S., Lippman S.I., Zhao X., Broach J.R. (2008). How *Saccharomyces* responds to nutrients. Annu. Rev. Genet..

[27] Johnson A., Skotheim J.M. (2013). Start and the restriction point. Curr. Opin. Cell Biol..

[28] Ewald J.C., Kuehne A., Zamboni N., Skotheim J.M. (2016). The yeast cyclin-dependent kinase routes carbon fluxes to fuel cell cycle progression. Mol. Cell.

[29] Turner J.J., Ewald J.C., Skotheim J.M. (2012). Cell size control in yeast. Curr. Biol..

[30] Bu P., Nagar S., Bhagwat M., Kaur P., Shah A., Zeng J. (2019). DNA damage response activates respiration and thereby enlarges dNTP pools to promote cell survival in budding yeast. J. Biol. Chem..

[31] Taylor S.D., Zhang H., Eaton J.S., Rodeheffer M.S., Lebedeva M.A., O'rourke T.W. (2005). The conserved Mec1/Rad53 nuclear checkpoint pathway regulates mitochondrial DNA copy number in *Saccharomyces cerevisiae*. Mol. Biol. Cell.

[32] Zhao X., Muller E.G., Rothstein R. (1998). A suppressor of two essential checkpoint genes identifies a novel protein that negatively affects dNTP pools. Mol. Cell.

[33] Young E.T., Dombek K.M., Tachibana C., Ideker T. (2003). Multiple pathways are co-regulated by the protein kinase Snf1 and the transcription factors Adr1 and Cat8. J. Biol. Chem..

[34] Biddick R.K., Law G.L., Young E.T. (2008). Adr1 and Cat8 mediate coactivator recruitment and chromatin remodeling at glucose-regulated genes. PLoS One.

[35] Tkach J.M., Yimit A., Lee A.Y., Riffle M., Costanzo M., Jaschob D. (2012). Dissecting DNA damage response pathways by analysing protein localization and abundance changes during DNA replication stress. Nat. Cell Biol..

[36] Li B., Reese J.C. (2001). Ssn6-Tup1 regulates *RNR3* by positioning nucleosomes and affecting the chromatin structure at the upstream repression sequence. J. Biol. Chem..

[37] Zhang Z., Reese J.C. (2005). Molecular genetic analysis of the yeast repressor Rfx1/Crt1 reveals a novel two-step regulatory mechanism. Mol. Cell Biol..

[38] Workman C.T., Mak H.C., McCuine S., Tagne J.-B., Agarwal M., Ozier O. (2006). A systems approach to mapping DNA damage response pathways. Science.

[39] Tsaponina O., Barsoum E., Astrom S.U., Chabes A. (2011). Ixr1 is required for the expression of the ribonucleotide reductase Rnr1 and maintenance of dNTP pools. PLOS Genet..

[40] Travesa A., Kuo D., de Bruin R.A.M., Kalashnikova T.I., Guaderrama M., Thai K. (2012). DNA replication stress differentially regulates G1/S genes via Rad53-dependent inactivation of Nrm1. EMBO J..

[41] Bastos de Oliveira F.M., Harris M.R., Brazauskas P., de Bruin R.A.M., Smolka M.B. (2012). Linking DNA replication checkpoint to MBF cell-cycle transcription reveals a distinct class of G1/S genes. EMBO J..

[42] Corcoles-Saez I., Ferat J.L., Costanzo M., Boone C.M., Cha R.S. (2019). Functional link between mitochondria and Rnr3, the minor catalytic subunit of yeast ribonucleotide reductase. Microb. Cell.

[43] Tsang C.K., Liu Y., Thomas J., Zhang Y., Zheng X.F. (2014). Superoxide dismutase 1 acts as a nuclear transcription factor to regulate oxidative stress resistance. Nat. Commun..

[44] Carter C.D., Kitchen L.E., Au W.C., Babic C.M., Basrai M.A. (2005). Loss of *SOD1* and *LYS7* sensitizes *Saccharomyces cerevisiae* to hydroxyurea and DNA damage agents and downregulates *MEC1* pathway effectors. Mol. Cell Biol..

[45] Zhou Z., Elledge S.J. (1993). *DUN1* encodes a protein kinase that controls the DNA damage response in yeast. Cell.

[46] Zhao X., Rothstein R. (2002). The Dun1 checkpoint kinase phosphorylates and regulates the ribonucleotide reductase inhibitor Sml1. Proc. Natl. Acad. Sci. U S A..

[47] Chen S.H., Smolka M.B., Zhou H. (2007). Mechanism of Dun1 activation by Rad53 phosphorylation in *Saccharomyces cerevisiae*. J. Biol. Chem..

[48] Kim K.Y., Truman A.W., Levin D.E. (2008). Yeast Mpk1 mitogen-activated protein kinase activates transcription through Swi4/Swi6 by a noncatalytic mechanism that requires upstream signal. Mol. Cell. Biol..

[49] Cucchi D., Gibson A., Martin S.A. (2021). The emerging relationship between metabolism and DNA repair. Cell Cycle.

[50] Moretton A., Loizou J.I. (2020). Interplay between cellular metabolism and the DNA damage response in cancer. Cancers (Basel).

[51] Shimizu I., Yoshida Y., Suda M., Minamino T. (2014). DNA damage response and metabolic disease. Cell Metab..

[52] Simpson-Lavy K.J., Bronstein A., Kupiec M., Johnston M. (2015). Cross-talk between carbon metabolism and the DNA damage response in *S. cerevisiae*. Cell Rep.

[53] Ferrari E., Bruhn C., Peretti M., Cassani C., Carotenuto W.V., Elgendy M. (2017). PP2A controls genome integrity by integrating nutrient-sensing and metabolic pathways with the DNA damage response. Mol. Cell.

[54] Pijuan J., María C., Herrero E., Bellí G. (2015). Impaired mitochondrial Fe-S cluster biogenesis activates the DNA damage response through different signaling mediators. J. Cell Sci..

[55] Cosentino C., Grieco D., Costanzo V. (2011). ATM activates the pentose phosphate pathway promoting anti-oxidant defence and DNA repair. EMBO J..

[56] Li L., Wang J., Yang Z., Zhao Y., Jiang H., Jiang L. (2022). Metabolic remodeling maintains a reducing environment for rapid activation of the yeast DNA replication checkpoint. EMBO J..

[57] Bhagwat M., Nagar S., Kaur P., Mehta R., Vancurova I., Vancura A. (2021). Replication stress inhibits synthesis of histone mRNAs in yeast by removing Spt10p and Spt21p from the histone promoters. J. Bio.l Chem..

[58] Guo Z., Kozlov S., Lavin M.F., Person M.D., Paull T.T. (2010). ATM activation by oxidative stress. Science.

[59] Paull T.T. (2015). Mechanisms of ATM activation. Annu. Rev. Biochem..

[60] Ho B., Sanford E.J., Loll-Krippleber R., Torres N.P., Smolka M.B., Brown G.W. (2023). Mec1-independent activation of the Rad53 checkpoint kinase revealed by quantitative analysis of protein localization dynamics. Elife.

[61] Ajazi A., Choudhary R., Tronci L., Bachi A., Bruhn C. (2022). CTP sensing and Mec1ATR-Rad53CHK1/CHK2 mediate a two-layered response to inhibition of glutamine metabolism. Plos Genet..

[62] Fendt S.M., Sauer U. (2010). Transcriptional regulation of respiration in yeast metabolizing differently repressive carbon substrates. BMC Syst. Biol..

[63] Liu Z., Butow R.A. (2006). Mitochondrial retrograde signaling. Annu. Rev. Genet..

[64] Liao X.S., Small W.C., Srere P.A., Butow R.A. (1991). Intramitochondrial functions regulate nonmitochondrial citrate synthase (*CIT2*) expression in *Saccharomyces cerevisiae*. Mol. Cell Biol.

[65] Yi C., Tong J., Lu P., Wang Y., Zhang J., Sun C. (2017). Formation of a Snf1-Mec1-Atg1 module on mitochondria governs energy deprivation-induced autophagy by regulating mitochondrial respiration. Dev. Cell.

[66] Gunjan A., Verreault A. (2003). A Rad53 kinase-dependent surveillance mechanism that regulates histone protein levels in S. cerevisiae. Cell.

[67] Singh R.K., Kabbaj M.H., Paik J., Gunjan A. (2009). Histone levels are regulated by phosphorylation and ubiquitylation-dependent proteolysis. Nat. Cell Biol..

[68] Pokholok D.K., Zeitlinger J., Hannett N.M., Reynolds D.B., Young R.A. (2006). Activated signal transduction kinases frequently occupy target genes. Science.

[69] Sheu Y.J., Kawaguchi R.K., Gillis J., Stillman B. (2022). Prevalent and dynamic binding of the cell cycle checkpoint kinase Rad53 to gene promoters. Elife.

[70] Bruhn C., Ajazi A., Ferrari E., Lanz M.C., Batrin R., Choudhary R. (2020). The Rad53CHK1/CHK2-Spt21NPAT and Tel1ATM axes couple glucose tolerance to histone dosage and subtelomeric silencing. Nat. Commun..

[71] Eriksson P.R., Ganguli D., Nagarajavel V., Clark D.J. (2012). Regulation of histone gene expression in budding yeast. Genetics.

[72] Kurat C.F., Recht J., Radovani E., Durbic T., Andrews B., Fillingham J. (2013). Regulation of histone gene transcription in yeast. Cell. Mol. Life Sci..

[73] Su C., Gao G., Schneider S., Helt C., Weiss C., O'Reilly M.A. (2004). DNA damage induces downregulation of histone gene expression through the G_1_ checkpoint pathway. EMBO J..

[74] Libuda D.E., Winston F. (2010). Alterations in DNA replication and histone levels promote histone gene amplification in Saccharomyces cerevisiae. Genetics.

[75] Chabes A., Stillman B. (2007). Constitutively high dNTP concentration inhibits cell cycle progression and the DNA damage checkpoint in yeast *Saccharomyces cerevisiae*. Proc. Natl. Acad. Sci. U S A..

[76] Kaur P., Nagar S., Mehta R., Sahadeo K., Vancura A. (2023). Hydroxyurea and inactivation of checkpoint kinase *MEC1* inhibit transcription termination and pre-mRNA cleavage at polyadenylation sites in budding yeast. Sci. Rep..

[77] Sherman F. (1991). Getting started with yeast. Methods Enzymol.

[78] Galdieri L., Vancura A. (2012). Acetyl-CoA carboxylase regulates global histone acetylation. J. Biol. Chem..

[79] Rosebrock A.P. (2017). Analysis of the budding yeast cell cycle by flow cytometry. Cold Spring Harb Protoc.

[80] Galdieri L., Zhang T., Rogerson D., Vancura A. (2016). Reduced histone expression or a defect in chromatin assembly induces respiration. Mol. Cell. Biol..

[81] Zhang T., Bu P., Zeng J., Vancura A. (2017). Increased heme synthesis in yeast induces a metabolic switch from fermentation to respiration even under conditions of glucose repression. J. Biol. Chem..

